# Inferior Pedicle Reduction Mammoplasty as Corrective Surgery after Breast Conserving Surgery and Radiation Therapy

**DOI:** 10.3390/jpm12101569

**Published:** 2022-09-23

**Authors:** Giulia Atzori, Simonetta Franchelli, Marco Gipponi, Chiara Cornacchia, Raquel Diaz, Francesca Depaoli, Federica Murelli, Marco Sparavigna, Piero Fregatti, Daniele Friedman

**Affiliations:** 1 Breast Surgery Clinic, San Martino Policlinic Hospital, 16132 Genoa, Italy; 2 Department of Surgical Sciences and Integrated Diagnostic (DISC), School of Medicine, University of Genoa, 16132 Genoa, Italy

**Keywords:** reduction mammoplasty, oncoplastic breast-conserving surgery, breast cancer, BREAST-Q

## Abstract

Background/Aim—Twenty patients had corrective reconstruction surgery by means of a reduction mammaplasty or mastopexy after a previous BCS (Breast Conserving Surgery) and RT (Radiation Therapy); the risk factors and post-operative complications were reported in order to define a safe and effective technique for reduction mammaplasty in previously irradiated breast cancer patients. Materials and Methods—From June 2011 to December 2019, 20 pts. were operated on at the Breast Surgery Clinic of San Martino Policlinic Hospital, Genoa, Italy. Pre- and post-operative parameters included clinic-pathological features of the primary tumor; a lapse of time from primary radio-surgery; the extent of follow-up; the rate of post-operative wound infections; the persistence of breast asymmetry, and a post-operative patient satisfaction index by means of a BREAST-Q questionnaire. Results—Three patients (15%) developed minor complications in the irradiated breast, but no complication was observed into the non-irradiated breast. No statistically significant correlation was found between the post-operative complications and the risk factors. The statistical analysis of BREAST-Q questionnaire responses gave an average patient’s satisfaction index that was equal to 90.8/100 (range: 44 to 100). Conclusions—Inferior pedicle reduction mammoplasty is an effective reduction mammoplasty technique in regard to the extent of breast tissues that are to be removed both in irradiated and contralateral breast; moreover, the incidence of post-operative complications is clearly limited when a careful technique is adopted, and it can be reasonably applied also in patients with co-morbidity factors.

## 1. Introduction

Breast-conserving surgery (BCS) with radiation therapy (RT) is a reliable alternative to mastectomy and it is widely adopted usually with good cosmetic results [1]. For this reason, more and more patients are presently undergoing BCS, although this has led to an increased number of patients who are referred at a later stage to the plastic surgeon for correcting asymmetry or seeking a breast reduction. As a matter of fact, patients with a large and ptosic breast should have primarily benefited from oncoplastic breast conserving surgery (OBCS) with synchronous contralateral reduction mammaplasty, but either this option was not proposed, or it was refused due to oncological safety. Other patients have developed breast asymmetry as a result of weight gain over the years, adjuvant hormone therapy, or RT-induced fibrosis.

The plastic surgeon has, therefore, to deal with irradiated tissues, with the inherent higher risk of post-operative complications with less-than-optimal cosmetic outcome [2,3]. This has limited the compliance with this type of plastic reconstruction and, consequently, there is a paucity of data regarding corrective surgery in the irradiated breast. However, notwithstanding these criticisms, such corrective surgery can be performed while one is taking care of a few precautions. Herein, our experience on 20 patients that were undergoing reduction mammaplasty or mastopexy after previous BCS and RT is reported; the risk factors and post-operative complications are reported in order to define a safe and effective technique for reduction mammaplasty in previously irradiated breast cancer patients.

## 2. Patients and Methods

A retrospective study was performed in 20 patients that were undergoing from June 2011 to December 2019 either reduction mammaplasty or mastopexy after a previous BCS with an adjuvant RT at the Breast Surgery Clinic of San Martino Policlinic Hospital, Genoa, Italy. Data were obtained from a prospective institutional database. All of the patients were referred to the plastic surgeon to correct their breast hypertrophy or severe ptosis with asymmetry as well as breast distortion.

The preoperative parameters included: age; body mass index (BMI); hypertension; diabetes; smoking habit; histologic and biologic factors regarding the primary tumor (tumor histotype and phenotype, tumor site, date and type of BCS); previous cumulative radiation dose including tumor bed boost (mean: 42 Gy; range 31–48 Gy); previous hormone therapy and/or chemotherapy, and plastic surgery parameters (type of surgery and weight of tissue removed). The post-operative data included: the length of follow-up; the rate of post-operative wound infections; the persistence of breast asymmetry, and the post-operative patient satisfaction index that was assessed by means of the validated BREAST-Q questionnaire [4]. The BREAST-Q is a rigorously developed patient-reported outcome measure designed to assess outcomes among women who are undergoing different types of breast surgery from the patient’s perspective. It was developed according to Rash’s model; the Breast-Q provides a score that is on a scale of 0 to 100, with a higher value indicating a greater satisfaction that refers to the patient’s perceptions of the results of surgery. The results of this satisfaction are reported in percentage values. The questionnaire was administered one year after the plastic surgery procedures.

With regard to the plastic surgical planning, even patients with co-morbidities might be suitable for corrective surgery; the volume and shape of the breast, the nipple position, and the grade of the breast ptosis were evaluated to define the best surgical option. Antibiotics prophylaxis was always performed with perioperative Cefazolin 14–20 mg/kg once; Clindamycin 600 mg was used in patients with a reported allergy to penicillin. All of the patients were informed about the surgical complications, and they gave their written consent for it; moreover, as recommended by the First International Conference of Standardization of Oncoplastic Breast Conserving Surgery, they got photographic documentation before and after the surgery [5].

Usually, the patients were discharged on the third post-operative day, without drains, and they were followed-up in the outpatient clinic once weekly for about one month, and after that, at 3, 6, and 12 months, and then, yearly. All of the complications such as infections, flap or nipple areola complex (NAC) loss, and persistent asymmetry that required re-operation were defined as major complications. Prolonged edema, wound dehiscence, partial NAC suffering, and liponecrosis were considered minor complications. An oncologic follow-up included a breast clinical evaluation and an annual bilateral mammography. As for the statistical analysis, the Chi-square test was applied to assess the correlations between the risk factors and the post-operative complications.

### Surgical Technique

Each operation was performed by S.F., an experienced Plastic Surgeon with more than 20 years of experience. Preoperative planning was made with the patient in a standing position, and preoperative pictures were contextually taken. The surgery consisted of a bilateral mastopexy or a bilateral reduction mammaplasty using an inferior pedicle technique in a standard fashion. The base of the inferior pedicle ranged from 5 to 10 cm. In an irradiated breast, the amputation and the grafting of the nipple-areola complex (NAC) were performed whenever the distance of the NAC from its actual to its ideal position (21–23 cm) was >7 cm; on the contralateral breast the NAC rising ranged from 1.5 cm to 12 cm. In order to achieve the better preservation of the dermal vascular plexus-limiting ischemia of the radiated flaps, great care was taken in underlying the skin flaps whose thickness was about 1.5 cm. A drain was placed before the closure of the wounds in each breast.

## 3. Results

Demographic and oncological data are reported in Table 1; briefly, the mean age was 57 (range: 38–73 years) and the average BMI was 25.8 (range: 22–34 kg/m^2^). Ten patients had no risk factors, six patients had at least one risk factor, while two or more risk factors were present in four patients; hypertension was the most common co-morbidity factor (40%), which was followed by a smoking habit (25%), while only one patient had diabetes (5%). The corrective surgery of the irradiated breast included an inferior pedicle reduction mammoplasty (IPRM) in 14 patients, and a mastopexy in six patients. An amputation and NAC grafting was performed in four patients. The mean weight of resected gland was 259.5 g (range: 0–1000); the mean lapse of time from the BCS to the corrective plastic surgery was 72 months (range: 12–264 months). The median follow-up after the corrective surgery was 54.7 months (range: 18–119 months).

**Figure 1:**
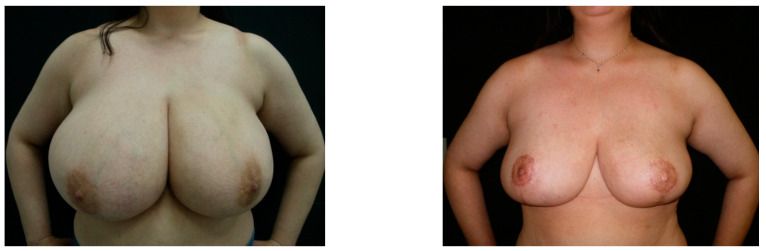
Pre- (**left** side) and post-operative (**right** side) images of a patient who had undergone a reduction mammoplasty of the irradiated tissue, with them having experienced a post-operative weight loss of nearly 30 kg.

With regard to the post-operative complications, only minor complications (*n* = 3; 15%) were reported in the irradiated breasts, including a prolonged breast edema (*n* = 2) and a wound dehiscence with marginal and superficial necrosis of the cutaneous flaps (*n* = 1). Conversely, no complication was observed in the non-irradiated breasts. No statistically significant correlation was found between the post-operative complications and the risk factors. With regard to the patient satisfaction index, most of patients were clearly satisfied at their post-operative assessment, and the statistical analysis of the BREAST-Q questionnaire responses gave an average patient’s satisfaction index that was equal to 90.8/100 (range: 44 to 100). At the most recent follow-up visit, the breast asymmetry (difference of volume of one breast compared to the contralateral) was observed in three patients due to a significant weight gain which occurred several months after the surgery. Conversely, no cosmetic impairment was observed in the patient with a weight loss of nearly 30 kg (Figure 1).

## 4. Discussion

BCS procedures are more and more frequently performed in patients with early breast cancer, although the need to achieve satisfactory aesthetic outcomes and to improve the quality of life of these patients has led most surgeons to develop OBCS. Since its introduction, the combination of the principles of surgical oncology and plastic surgery have clearly improved the cosmetic outcomes whenever a large amount of breast tissue needs to be removed [6]. OBCS can achieve more acceptable aesthetic outcomes when it is compared to those of BCS by reducing breast size, minimizing the impact of radiotherapy in large breasts, and achieving a preferred breast size and shape [7]. Poor cosmetic outcomes after BCS can be predicted by there being a certain percentage of breast tissue that is removed and by the tumor location and, in such cases, OBCS should be primarily recommended [2,8].

Reduction mammoplasty is frequently performed in patients with macromastia or severe ptosis but, less frequently, in previously irradiated breast because mammoplastic reduction or mastopexy in radio-treated tissues is challenging for the plastic surgeon. Actually, the early and late effects of RT on breast tissues were highlighted by different authors [9,10,11,12]. Radiation damage usually develops within four to 12 months after RT, and it can progress over several years. The early effects that it can have, such as inflammation and edema up to de-epithelialization, are to be carefully considered before any type of surgery is conducted. For this reason, we always suggest at least an 18-month waiting period before any type of reduction mammaplasty, just to avoid acute skin inflammation [3]. Late tissue changes due to fibrosis increases the complication rate (up to 54%) such as: prolonged edema; infection; wound dehiscence; NAC suffering; liponecrosis, and flap necrosis [13,14]. Moreover, post-operative complications increase with concomitant co-morbidity factors such as: obesity; hypertension; smoking habits; advanced age; and removal of a large amount of glandular tissue [3,15]. This higher risk is likely related to the presence of fibroblasts and stem cell shortfall which are caused by RT; actually, irradiated tissues demonstrate less ability to respond to surgical injury and tension on wound closures [16].

The higher risk of post-operative complications coupled with limited literature data are currently limiting plastic surgeons to propose corrective options in previously irradiated breast; as a matter of fact, only a few articles and two reviews on this topic are available in the literature, each with limiting and controversial findings. First, only a small number of patients were assessed, with there being a mean number of 12 patients (range: 1–39 pts) [3,17]. Moreover, there was a wide variation in the post-operative complications, ranging from 7.5% to 54%, and they were most likely related to obesity, smoking habits, advanced age, and removal of a large amount of glandular tissue. This led some authors to exclude patients with risk factors, therefore, the outcome analysis is further challenged [16,17]. Other questionable aspects regard the use of different reduction mammaplasty techniques, sometimes in the same patients, so that any technique comparison is not assessable [8,15,18,19].

**Figure 2:**
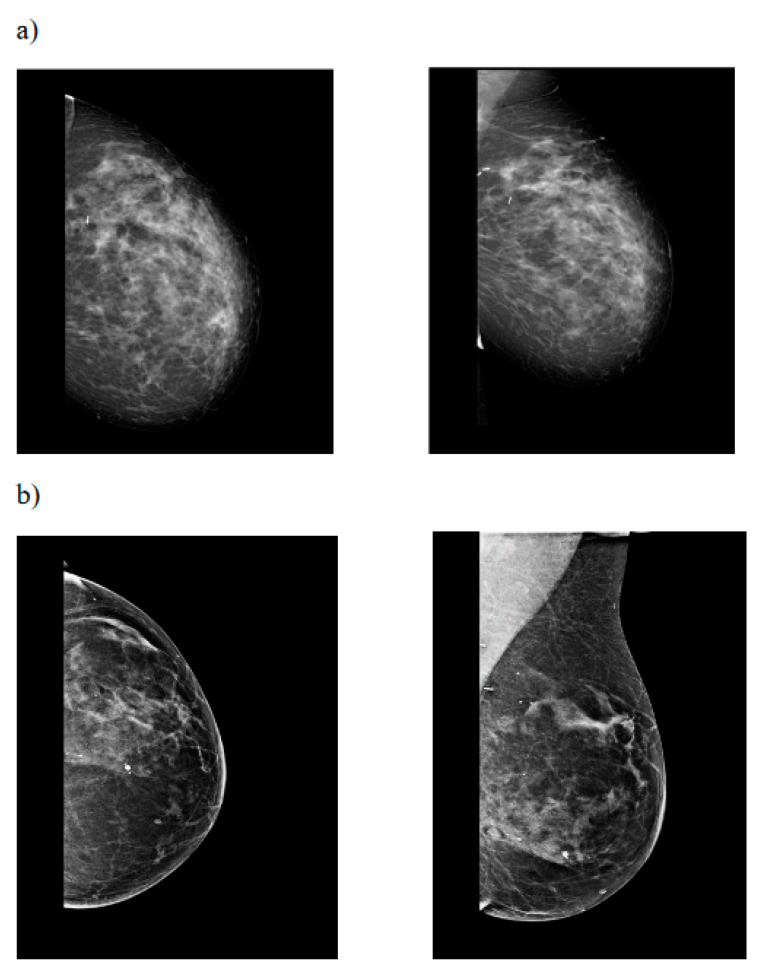
Cranio-caudal (**left** side) and medio-lateral oblique (**right** side) projection of left breast; (**a**) preoperative imaging (**b**) post-operative imaging. No architectural change and/or post-operative breast tissue modification is appreciable.

To our knowledge, our clinical series represents a peculiar experience for different reasons: in a cohort of 20 patients, the same IPRM technique was systematically applied by the same plastic surgeon; all of the patients were followed by the same plastic surgeon, and photographic documentation was acquired at each visit. Patients with risk factors were included, unless for anesthesiologic requirements, so that their correlation with the post-operative complications could be investigated. In our opinion, the use of the IRMR technique with a skin flap of adequate thickness is probably the key feature of our series. As confirmed by van Deventer et al. [20] in their study regarding breast vascularization, IPRM increases the preservation of the largest amount of perforators that are coming from the internal thoracic artery and anterior intercostal arteries [20]. In an irradiated breast, a wider pedicle with a dual-blood supply becomes crucial to ensure good vascularization in order to reduce the post-operative complications, such as liponecrosis. The inferior pedicle can be harvested with variable width, relative to the amount of breast tissue that is to be removed, thus leading to good cosmetic results, even in large and ptotic breasts without limitations about what tissue is to be removed (Figure 1) [21,22]. Moreover, it can be applied either in the irradiated or contralateral breast thus ensuring that there is better symmetry, thereby avoiding visible residual scars or asymmetry due to the application of different techniques of OBCS, as is reported by other authors [22]. The preservation of blood supply to the breast tissue could explain the lack of changes in the post-operative mammography of the residual irradiated breasts in our series (Figure 2a,b). Finally, the adequate thickness of reduced skin flaps ensures the preservation of the dermal vascular pattern, without adopting further prophylactic measures as is suggested by other authors [23,24].

In non-irradiated breasts, no difference exists between the superior and inferior pedicle techniques in terms of the cosmetic results and the complications, and the choice among the different techniques depends on the quantity of tissue that is to be removed and the distance of the NAC. It is worth noting that Kronowitz et al. [25] observed that the superior pedicle was associated with an increased complication rate when a reduction mammoplasty was performed in an irradiated breast, probably due to the complete undermining of the glandular tissues from the pectoralis muscle.

## 5. Conclusions

Our data suggest that IPRM is an effective reduction mammoplasty technique in regard to the extent of the breast tissue that is to be removed both in irradiated and contralateral breasts; moreover, the incidence of post-operative complications is clearly limited when a careful technique is adopted, and it can be reasonably applied, also, in patients with co-morbidity factors.

## Figures and Tables

**Table 1:** Clinical and pathological features.

	Breast Reduction *n* = 14	Mastopexy *n* = 6	Total *n* = 20	Correlation with Postoperative Complications
Mean age	56.5 years	58 years	57 years	
Mean BMI	26 kg/m^2^	25.4 kg/m^2^	25.8 kg/m^2^	
Smoking	4	1	5	no (*p* 0.781)
Hypertension	7	1	8	no (*p* 0.125)
Diabetes	1	0	1	no (*p* 0.666)
Upper tumor	11	4	15	
Lower-site tumor	3	2	5	
Ductal Carcinoma	12	5	17	
Other	2	1	3	
Luminal-type	11	3	14	
HER2/Triple-Negative	3	3	6	
Adjuvant chemotherapy	8	3	11	no (*p* 0.659)
Adjuvant hormonal therapy	10	6	16	no (*p* 0.531)
Average tissue removed	370.7 g	0	259.5 g	
Major complications	0	0	0	
Minor complications	3	0	3	
Patients satisfaction (yes)	12/14	6	18/20	
Average follow-up time	46.2 months	74 months	54.7 months	

## Data Availability

Data supporting reported results are available in Insititutional database.

## Abbreviations

BCS: breast conserving surgery; RT: radiation therapy; OBCS: oncoplastic breast conserving surgery; NAC: nipple areola complex; IPRM: inferior pedicle reduction mammoplasty.
